# Cancer across the tree of life: cooperation and cheating in multicellularity

**DOI:** 10.1098/rstb.2014.0219

**Published:** 2015-07-19

**Authors:** C. Athena Aktipis, Amy M. Boddy, Gunther Jansen, Urszula Hibner, Michael E. Hochberg, Carlo C. Maley, Gerald S. Wilkinson

**Affiliations:** 1 Center for Evolution and Cancer, University of California San Francisco, San Francisco, CA 94143, USA; 2 Department of Psychology, Arizona State University, Tempe, AZ 85287–4501, USA; 3 Centre for Evolution and Cancer, Institute for Cancer Research, 123 Old Brompton Road, London SW7 3RP, UK; 4 Department of Evolutionary Ecology and Genetics, University of Kiel, Am Botanischen Garten 1–9, 24118 Kiel, Germany; 5 CNRS, UMR 5535, Institut de Génétique Moléculaire de Montpellier, Université de Montpellier, Montpellier, France; 6 Institut des Sciences de l'Evolution, CNRS UMR5554, Université Montpellier, 34095 Montpellier, France; 7 Santa Fe Institute, 1399 Hyde Park Road, Santa Fe, NM 87501, USA; 8 Biodesign Institute, School of Life Sciences, Arizona State University, PO Box 8724501, Tempe, AZ 85287–4501, USA; 9 Department of Biology, University of Maryland, College Park, MD 20742, USA; 10 Institute for Advanced Study, Wissenschaftskolleg zu Berlin, Berlin, Germany

**Keywords:** complex multicellularity, cancer hallmarks, major transitions, microenvironment, division of labour, resource allocation

## Abstract

Multicellularity is characterized by cooperation among cells for the development, maintenance and reproduction of the multicellular organism. Cancer can be viewed as cheating within this cooperative multicellular system. Complex multicellularity, and the cooperation underlying it, has evolved independently multiple times. We review the existing literature on cancer and cancer-like phenomena across life, not only focusing on complex multicellularity but also reviewing cancer-like phenomena across the tree of life more broadly. We find that cancer is characterized by a breakdown of the central features of cooperation that characterize multicellularity, including cheating in proliferation inhibition, cell death, division of labour, resource allocation and extracellular environment maintenance (which we term the five foundations of multicellularity). Cheating on division of labour, exhibited by a lack of differentiation and disorganized cell masses, has been observed in all forms of multicellularity. This suggests that deregulation of differentiation is a fundamental and universal aspect of carcinogenesis that may be underappreciated in cancer biology. Understanding cancer as a breakdown of multicellular cooperation provides novel insights into cancer hallmarks and suggests a set of assays and biomarkers that can be applied across species and characterize the fundamental requirements for generating a cancer.

## 1. Introduction

Multicellularity requires the suppression of cell-level fitness in order to promote organism level fitness [1,2]. Cancer represents a breakdown of this multicellular cooperation, with cancer cells ‘cheating’ in ways that can have devastating effects for organism level fitness [3–5]. Effective multicellularity requires not just cooperation among cells but also mechanisms for suppressing conflict that results from mutations that can enhance cell-level fitness at the expense of the organism [5,6]. In other words, effective multicellularity requires the suppression of somatic cheating to some degree, and the cancer that results from that cheating.

In this review, we examine cooperation and cheating across multicellular life, focusing on cancer and cancer-like phenomena in complex multicellularity.^1^ Complex multicellularity has evolved independently at least seven times (once in Metazoa, once each in ascomycete and basidiomycete fungi, once in embryophytes, once in chlorophytes and at least once in both the rhodophytes and heterokontophytes) [7,8], meaning that multicellular cooperation and cheating suppression have also independently evolved many times. Most work characterizing the fundamental features of cancer and neoplastic growths has focused on cancer in humans and mice (e.g. [9,10]), with some work on cancer in captive animals [11,12], and very little work on cancer in the wild [13]. Here, we summarize reports of cancer and cancer-like phenomena in each of the seven branches of complex multicellularity in the tree of life. We find that cancer is characterized by a breakdown in the central features of cooperation that characterize multicellularity including cheating in proliferation inhibition, cell death, division of labour, resource allocation and extracellular environment maintenance (which we term the five foundations of multicellularity). We define cheating here as simply the breakdown of shared rules (including genetically encoded phenotypes or behaviours) that leads to a fitness advantage on the cellular level for the cheater. We do not imply that cheating is a pre-adapted or evolved ‘strategy’, but is rather a functional manifestation as described below.

## 2. The five foundations of multicellular cooperation

**Figure 1.**
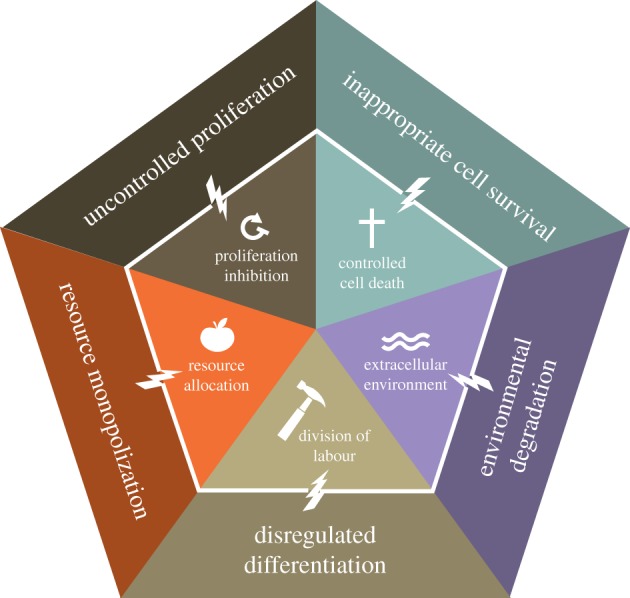
The five foundations of multicellularity. Effective multicellularity requires several types of cooperation: proliferation inhibition, controlled cell death, resource allocation, division of labour, and creation and maintenance of the extracellular environment. These cooperative cell behaviours were selected during the evolution of multicellularity and enable higher level function of the multicellular body. When the traits that make up the foundation of multicellular cooperation break down, this leads to uncontrolled proliferation, inappropriate cell survival, resource monopolization, deregulated differentiation and degradation of the environment. These cheating phenotypes are characteristic of cancer.

Multicellularity is characterized by cooperation among cells, tissues and when present, organ systems, for the development, maintenance and reproduction of the multicellular organism. The underlying processes favouring the transition from unicellular to multicellular organisms [14] are derived from some of the foundations of cooperation theory [15,16]. Multicellularity evolved because the formation of groups of cells with new physiological and behavioural capacities provided advantages over some forms of unicellular living [1,2]. The five most important novelties, which we here term the five foundations of multicellularity (figure 1), involve cell-level cooperative capacities [5]: (i) inhibiting cell proliferation [1], (ii) regulation of cell death [17–19], (iii) division of labour [1,6], (iv) resource transport [7,20] and (v) creation and maintenance of the extracellular environment [21,22].

Cheating in these domains of cooperation can lead to a breakdown of multicellular function (figure 1). Over evolutionary time, selection pressures on multicellularity have therefore led to mechanisms that suppress forms of cheating that negatively impact organismal fitness [6]. In this section, we describe how a breakdown of cooperation in each foundation of multicellularity manifests in terms of cancer and cancer-like features. Using this framework, we then review cancer across life.

### (a) Proliferation inhibition

Control over proliferation is necessary for functional multicellularity, allowing for development, tissue maintenance and many other functions. With the exception of specialized stem cells and their immediate descendants as well as a few highly regenerative tissues such as the liver, the capacity of proliferation is strictly suppressed, even in high-turnover tissues such as mammalian skin or intestine. To control proliferation, multicellular organisms have evolved redundant checks on the cell cycle and mechanisms that automatically trigger apoptosis or senescence when cells start to proliferate [23]. These systems thus suppress somatic cheating owing to uncontrolled cell proliferation. A lack of proliferation inhibition leads to uncontrolled cell replication, considered as one of the central features of cancer [24]. It encompasses several other hallmarks of cancer: evading growth suppression, sustaining proliferative signalling and enabling replicative immortality [10].

### (b) Controlled cell death

In multicellular organisms, programmed cell death (PCD) is a central contributor to the development, organization and maintenance of the body [17], allowing for embryonic development and tissue maintenance [18,19]. The evolutionary origin of PCD in multicellular organisms can be traced back to similar functions in unicellular organisms [25–27] that were mostly responsible for defence against infections [28,29]. PCD allows elimination of cells with less functional phenotypes [30], sculpting of structures [31], precise coordination of numbers of different cell types and their functional connections [32], and elimination of obsolete tissues [33]. In an adult, it provides a sensitive mechanism for elimination of danger that might come from infection, malformation or uncontrolled expansion of cells [34]. PCD is a central mechanism of tumour suppression [23,35] and resistance to PCD is recognized as one of the hallmarks of cancer [9,10].

### (c) Division of labour

One of the key features of complex multicellularity is the diversity of tissue and cell types [36] that allows different functions to be simultaneously performed in a single body. Effective multicellularity requires cells in particular tissues to perform specific functions [1], which enables division of labour [37]. Division of labour has been shown to evolve spontaneously in models of the evolution of multicellularity [38]. The process of differentiation that generates those cell types must be properly controlled during multicellular development and regulated for effective tissue maintenance. Inappropriate tissue differentiation is often regarded as a central feature of neoplasms and cancer [39], with the grading of severity of tumours being based on the degree of differentiation that remains in the tissue. However, a lack of appropriate differentiation is surprisingly not included in the traditional hallmarks framework (see Discussion) [9,10].

### (d) Resource allocation and transport

Cells require resources to survive and perform their functions. Larger multicellular aggregations require systems of resource transport because cells on the interior cannot meet their oxygen and nutrient requirements through diffusion alone [7,40]. Indeed, transfer of resources from high- to low-resource sites has been shown to provide an advantage for cell clustering in models of the evolution of multicellularity [20]. Systems of resource transport are thus central aspects of multicellular cooperation [7,41]. Some multicellular organisms, such as chordates and embryophytes, possess the capacity for bulk transport through a branching vascular system, while other forms of multicellularity indirectly or directly transport resources through interior cavities. The simplest system, gap junctions, is found in cnidarians [7]. Furthermore, multicellular organisms typically employ a more efficient and less wasteful metabolism than single-celled organisms [42]. Disruption of or manipulation of resource transport systems are central characteristics of cancer, as angiogenic signalling (i.e. inducing the growth of blood vessels) and deregulated metabolism are considered cancer hallmarks [9,10]. Thus, effective cancer suppression requires regulation of resource monopolization and metabolic pathways.

### (e) Extracellular environment maintenance

Multicellularity requires not only equitable resource allocation and labour performance from cells, but also creation and maintenance of a shared environment. Waste produced by normally functioning cells in a multicellular body needs to be cleared, and dead cells need to be identified and properly recycled. Moreover, cells need to maintain the extracellular matrix [22], which is made up of networks of proteins that form supporting structures, such as basement membranes [21]. Cancer cells destroy the extracellular matrix using a variety of factors (e.g. matrix metalloproteinases), thus facilitating cell invasion [43,44], one of the hallmarks of cancer [9,10]. Cancer cells also destroy the extracellular matrix as a result of by-products of glycolytic metabolism [45]. Destruction of the extracellular environment is such a central feature of cancer that the genetic capacities underlying it have been collectively termed the ‘cancer degradome’ [46]. Finally, in cancer the immune response, which normally identifies and removes invaders, is often co-opted to enhance tumour growth through inflammation [47,48] (box 1).

Box 1. **Definitions.**

**Tumour.** An abnormal mass, which may or may not be cellular (e.g. fibroid masses).

**Malformation**. A morphological defect resulting from an abnormal developmental process, i.e. teratogenesis.

**Neoplasm**. A mass of cells without physiological function, typically involving hyperproliferation and the disruption of normal tissue organization.

**Lesion**. Abnormality in the tissue, often characterized by lack of or inappropriate cell differentiation.

**Hyperplasia**. An increase in the number of cells, often appearing as a mass.

**Carcinoma**. A cancer that originates in an epithelial cell.

**Metastasis**. The spread of cancer from its primary site, often from one tissue or organ to another.

**Micrometastasis**. Metastases that are too small to be detected with current imaging.

**Cancer**. A neoplasm that has invaded through basal membrane boundaries or metastasized into locations distant from the initial site of the neoplasm.

**Cooperation**. Transmission of benefits and/or coordination of actions that augments fitness of the larger ensemble (e.g. organism) and/or facilitates shared goals.

**Cheating**. Breaking of shared rules, including genetically encoded phenotypes or behaviours, that leads to a fitness advantage for the cheater.

## 3. Survey of cancer across life

**Figure 2.**
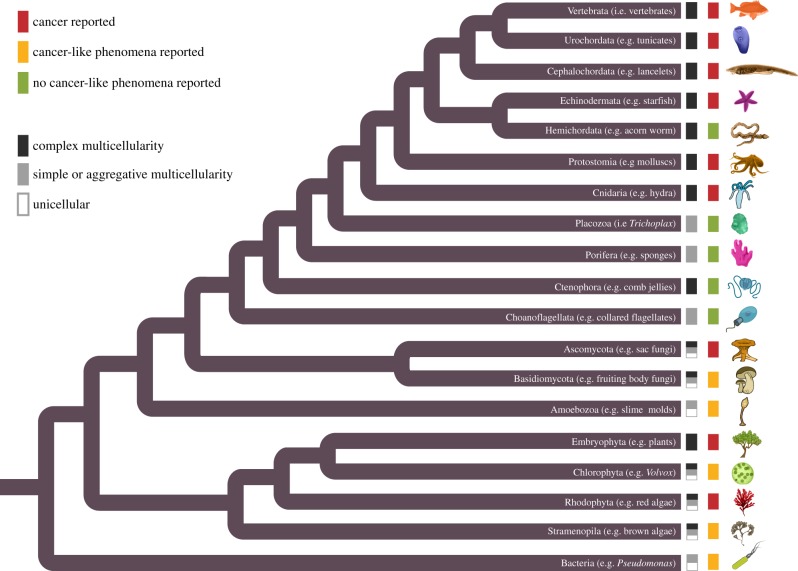
Cancer across the tree of life. Phylogenetic relationships among the organisms discussed in the paper inferred from previous published trees [7,8,49–51]. This figure includes all lineages containing multicellular forms [7,8] but is not meant to denote ancestral states or all possible independent origins. Black, grey or white boxes at branch tip indicates cellularity as unicellular (white), simple or aggregative multicellularity (grey) or complex multicellularity (black) in extant species [7,8]. Red, yellow or green coloured boxes represent whether a cancer phenotype (invasion or metastasis) was reported in the literature for that lineage (red box), a cancer-like observation (abnormal proliferation or differentiation)—such as callus or galls (yellow box) or no cancer-like phenotype has been described in the literature (green box).

Viewing cancer as a phenomenon of cheating on the cooperation that characterizes multicellular organisms generates predictions that can be tested by surveying independent multicellular lineages. Larger and more complex forms of multicellularity (based on the probability per cell of mutations and the fact that complex multicellularlity requires more complex regulatory networks that can be damaged) should have both greater susceptibility to cancer and more mechanisms for cancer suppression. Rather than focus exclusively on cancer as clinically defined for humans (i.e. characterized by invasion and metastasis), we consider cancer-like phenomena more broadly, which include neoplastic growths characterized by abnormal proliferation and differentiation (hereafter, we refer to ‘cancer-like’ as ‘cancer’). In this section, we describe what is known about cancer-like phenomena using examples from each lineage with complex multicellularity. We also discuss related taxa showing simple multicellularity, such as sponges, and aggregative multicellularity, such as cellular slime moulds and bacterial biofilms. Within each of the seven independent branches of complex multicellularity (figure 2) [7,8]: animals (metazoans), fungi (both ascomycetes and basidiomycetes), green algae (embryophytes and chlorophytes), red algae (rhodophytes) and brown algae (phaeophytes), we examine the extent to which cancer susceptibility and suppression are characterized by cheating and cooperation in the foundations of multicellularity: proliferation inhibition, cell death, division of labour, resource allocation and environment maintenance.

### (a) Animals: Metazoa

#### (i) Deuterostome chordates

Cancers have been observed across virtually all vertebrates, regardless of body size and lifespan [11,52]. Based on over 9000 necropsies, there is evidence that birds and reptiles have lower cancer rates than mammals (1.9% of necropsies in birds, 2.2% in reptiles and 2.8% in mammals) [52]. Larger and longer-lived animals, such as whales and elephants, have lower cancer rates than would be expected given the number of cells and number of cell divisions that occur [11], a phenomenon termed Peto's paradox (see Discussion section).

Two species of vertebrates stand out as having little if any cancer: naked mole rats and blind mole rats. There have been over 380 necropsies performed on naked mole rats from laboratory colonies with no observed malignant neoplasms [53,54], though there is evidence of pre-malignant neoplasms [54]. Additionally, blind mole rats have been studied for over 50 years in the laboratory without any case of spontaneous tumour formation [55,56].

Tumours have been reported from all three chordate subphyla. There are reports in urochordates, i.e. tunicates [57,58], which include elongated protrusions on the body [59], both hypertrophy and hyperplasia in gut tissue [60], and nodules induced by bacteria [61]. Gut lesions were associated with a sporozoan infection but persisted after the removal of the parasite. An irregular mass of tissue was found in the midgut of an adult amphioxus, a cephalochordate, and described as a chromaffinoma [62]. The tumour masses and hyperplasias are indicative of cheating on cell proliferation, death or both.

#### (ii) Deuterostome invertebrates

Twenty-four different types of neoplastic growth have been reported in echinoderms [63], a phylum which includes starfish, sea urchins, sand dollars and sea cucumbers. One frequently cited and sometimes disputed example involves pigmented lesions near the base of the arm in 7 of 95 brittle stars, *Ophiocomina nigra*, collected near Plymouth, UK [64]. Four specimens had two or more lesions, each of which consisted of a mass of abnormal melanocytes. Upon re-examination, Sparks [65] concluded that the lesions exhibit characteristics of neoplastic growth, including early stages comprised of embryonic melanocytes, growth by rapid cell division and evidence of epidermal spread, suggesting that some might even be malignant. The rapid cell division suggests those cells are cheating on the constraints of proliferation inhibition, and their embryonic state suggests loss of differentiation and thus cheating on division of labour. Their epidermal spread suggests cheating in extracellular environment maintenance.

A second example involves calcified protuberances on the aboral surface of sand dollars, *Echinarachnius parma*, which have been interpreted as a neoplastic disease that originated in the test sclerocytes [66]. Finally, a tumour was reported from the intestine of a sea cucumber, *Holothuria leucospilata* [67], although some authors [57,68] have subsequently dismissed this case as simply an unusual outgrowth of the gut. More recent cases of echinoderm tumours have not been reported, leading many to conclude that echinoderms have a low incidence of cancer-like growth [68–70]. Given that many species have the ability to regenerate arms [71] or organs [72] and some can live for more than 100 years [73], echinoderms may deserve increased study for potential anti-cancer properties.

Hemichordata is a small phylum that includes benthic marine organisms commonly called acorn worms, some of which have the ability to regenerate anterior structures, including nervous tissue [74]. We failed to find evidence of tumours in acorn worms, but they do possess copies of genes involved in cancer suppression, including three homologues of p53 [75]. In addition, cephalostatins, the most potent anti-tumour compounds so far found in marine organisms, were extracted from acorn worms [76].

#### (iii) Protostome invertebrates

Cancer-like growths have been reported for most protostome invertebrates. Spontaneously occurring benign and malignant tumours have been repeatedly reported for planarian flatworms [77–81]. Planarians have now become a model organism in ecotoxicology owing to their ability to form tumours after exposure to carcinogens, some of which can be rapidly destructive to the organism [82,83]. Reports of abnormal presence of mitotic figures imply proliferation inhibition cheating, and the formation of these tumour masses requires proliferation inhibition and/or controlled cell-death cheating. Their destructive nature implies maintenance of extracellular environment cheating and reports of undifferentiated cells in the tumours suggests division of labour cheating was also present. In the earthworm, *Lumbicus terrestris*, cold lesions can proliferate into epitheliomas [84]. Moreover, in both *Lumbricus* and *Eisenia*, myoblastomas were found to have been invaded by many blood vessels, and were able to penetrate the muscle fibres and destroy the surrounding epithelium [85]. The high density of blood vessels suggests resource allocation cheating and the destruction of the epithelium suggests maintenance of extracellular environment cheating. Other tumours have been described in older literature, but many of these could not be attributed conclusively to cancer (for a review of this literature, see [86]). Most convincing was a sipunculid marine worm with proliferating cells that obstructed the vascular tube and apparently disseminated to other tissues via the bloodstream.

Two types of lethal tumours, cancerous haemotocytes and germinomas, have been described in molluscs [87]. Both are characterized by atypical structures, many mitotic figures, rapid, invasive growth and metastasis [88]. Cancerous haemotocytes (previously described as leukaemia or haemic neoplasia) are caused by abnormally tetraploid, proliferating haemocytes of unclear origin. These cancerous cells overexpress the mitochondrial *hsp70* protein, which in turn leads to cytoplasmatic sequestration and inactivation of the tumour suppressor *p53* protein [89,90]. Transcriptomic analysis has recently revealed differential expression of cancer-related genes including *ras* and genes related to cell-cycle regulation, chromosome defects and apoptosis [91–93]. Cancerous haemotocytes have been reported for 15 bivalve species worldwide [94]. The disease can reach high prevalence (up to 95%) in some populations of softshell clam, *Mya arenaria*, particularly during autumn, whereas during the rest of the year prevalence remains around 10% [95]. Haematopoietic tumours in *M. arenaria* have been found at oil spill sites [96]. For an excellent review of this disease, including detailed prevalence data for multiple species and locations, see Barber [87].

Germinomas are neoplasms that originate in gonadal follicles, multiply until they fill the follicles and, after invading the surrounding connective tissue, spread to the body wall, genital ducts and the rest of the body. However, the disease does not appear lethal [97]. Germinomas appear to be examples of both proliferation cheating, as the cells multiply excessively, and also cheating of the extracellular environment maintenance, given the destruction of the connective tissue. Germinomas are rarely found in oysters, scallops or mussels, but in *M. arenaria* they can reach a prevalence of 3.3 to 50% [87,98,99].

Tumours have rarely been observed in crustaceans. A lymphosarcoma was found in haematopoietic tissue caused by hypertrophied, invasive, mitotically active and anaplastic lymphoid cells in the white shrimp [100]. The anaplasia, with loss of structural differentiation of mature cells, suggests division of labour cheating in these neoplasms, and the mitotic activity suggests proliferation inhibition cheating. Invasion, through its destruction of tissue membranes, involves cheating through destruction of the extracellular environment. Hypertrophy, or large cell size, may indicate resource monopolization cheating. Further findings include carcinomas in *Palaemon orientis* embryos [101] and in the red king crab hind gut [102], and a tumour-like abdominal lesion in brown shrimp [103] and in the lobster *Homarus americanus* [104].

In the arachnid *Phalangium opilio*, a proliferating prosomal tumour was observed that had pushed internal organs aside [86,105], indicating proliferation cheating. Moreover, an epithelial proliferation showing abnormal mitosis in the oviduct of *Pachygnatha clecki* was found to gradually invade the uterus [106], suggesting cheating through degradation of the extracellular environment.

Among insects, *Drosophila* flies have been widely exploited for studying the genetic causes of cancer and have assisted both in deciphering the genetic basis of human cancers and in identifying novel cancer genes. In larvae, neoplastic tumours do not respond to differentiation signals and may become invasive and immortal, suggesting division of labour, environment and cell-death cheating. Examples include epithelial tumours caused by activated RAS and mitochondrial dysfunction or polarity loss [107,108], myoblastomas, glial, neuroblast, haematopoietic and neuroepithelial tumours. Adults also show neuroblastomas, natural tumours in testes, follicles, brain, gut and malpighian tubules that can metastasize and kill the host [109–111]. A case of a thoracic tumour in a beetle was also documented [86].

Clearly, our survey of bilaterian animals indicates that all or nearly all are susceptible to cancer. There is evidence of all forms of somatic cheating including division of labour cheating with loss of differentiation, resource allocation cheating with angiogenesis, maintenance of environment cheating with invasion and destruction of tissue architecture, and proliferation inhibition cheating and controlled cell-death cheating with the generation of tumour masses and invasive/metastatic spread.

#### (iv) Cnidaria

**Figure 3.**
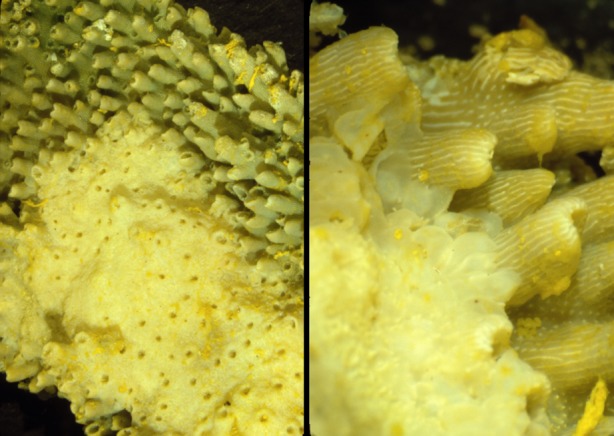
Cancer in corals. Corals often exhibit tumours called calicoblastic epitheliomas with loss of differentiation and destruction of the tissue architecture, including the mechanisms for resource allocation [113]. The normal tubular growth pattern in the upper right of both panels is being invaded by the relatively smooth, unstructured calicoblastic epithelioma. These samples are from the Grecian Rocks, Florida Keys (Florida Keys National Marine Sanctuary). The coral appears yellow because the samples were preserved in Helly's fixative which included potassium dichromate. Pictures courtesy of Esther Peters.

Cnidarians (e.g. sea anemones, corals, hydra and jellyfish) are a phylum with over 10 000 species [112] with relatively simple morphologies. Observations in both hydra and corals suggest that the cheating on multicellular cooperation that leads to cancer likely was a problem for some of the earliest metazoans. A variety of corals have been observed with cancer-like phenomena: smooth white tumours called calicoblastic epitheliomas that destroy the normal structure of the corals (figure 3) [113,114]. These neoplasms have a number of striking characteristics reminiscent of cancer in animals including rapid growth (proliferation inhibition cheating), loss of differentiation and specialized cells (division of labour cheating), loss of tissue architecture (environment maintenance cheating), proliferation of gastro-vascular canals (analogous to angiogenesis, resource allocation cheating), and are costly to the fitness of the organism as indicated by reduced fecundity [115]. Calicoblastic epitheliomas are relatively common. Thirty-nine per cent of massive *Porites* in the Philippines had such neoplasms [116].

In addition to the calicoblastic epitheliomas, necroses and abnormal growths can occur when two sufficiently different hydrocoral *Millepora dichotoma* individuals come in contact [117]. Furthermore, galls and other cancer-like growths can be generated in corals by infection with trematodes, fungi and green algae [115].

**Figure 4.**
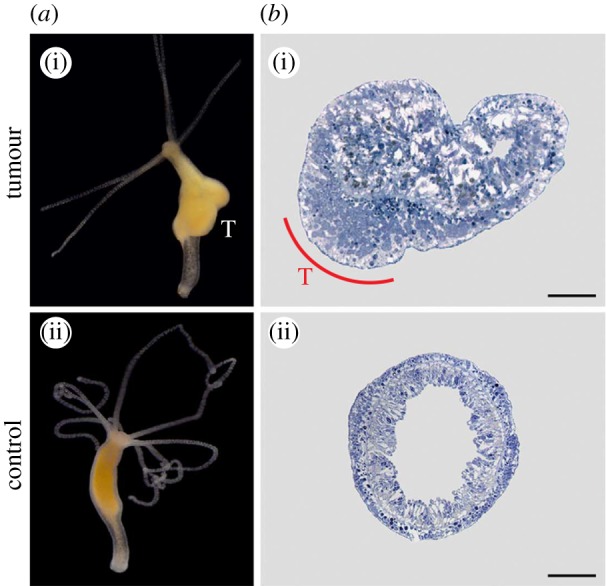
Cancer in hydra. Naturally occurring tumours have been found in *Hydra oligactis* and *Pelmatohydra robusta*. (*a*(i),*b*(i)) A tumour in *H. oligactis* (marked with a T) and (*a*(ii),*b*(ii)) normal controls (whole body and cross section). Adapted with permission from Domazet-Loso *et al.* [118].

Naturally occurring tumours in long-term cultures have recently been described in two species of *Hydra* and appear to develop from gamete tissue that fails to differentiate properly (figure 4) [118]. Transcriptome analysis revealed 196 misregulated genes, 44 of which have homology to tumour-related genes in mammals, and include genes affecting cell cycle, apoptosis, genomic stability and metabolism, suggesting at least the presence of proliferation inhibition cheating, controlled cell-death cheating and resource allocation cheating.

#### (v) Placozoa

The phylum Placozoa currently contains only one described species, *Trichoplax adhaerens*, which is considered to resemble a basal metazoan form [49]. *Trichoplax* is one of the most ‘simple’ multicellular organisms with only four cell types. It has an upper and lower epithelium that loosely surrounds fibre cells, with an irregular body shape and no symmetry or polarity. Reproduction is mainly asexual, but individuals can also reproduce sexually [119]. Trichoplax are thought to have efficient repair mechanisms. They can quickly regenerate after injuries or regenerate a complete individual from just a few cells. No definitive cancer-like phenotypes have been described and they have been observed to be resistant to radiation by X-rays [120].

#### (vi) Porifera

Sponges have no distinct tissues or organs. Instead, they use less specialized structures, such as pores, canals, ostia and chambers, for water, food and nutrient flow. Sponges form associations with diverse symbiotic microorganisms [121,122] and are subject to pathogenic diseases that cause necrosis [123]. They have innate immune systems with one of their defence mechanisms against pathogen damage being apoptosis [124]. However, some sponges are known to shed cells, at high rates under certain conditions [125]. This may be a mechanism to cull damaged or ageing cells. Sponges have no known cheating or cancer-like phenomena despite their long lifespans, though this may be due to a lack of study [126,127]. Moreover, certain antimitotic molecules have been isolated from sponges for potential therapeutic use in humans [128], but it is not known if the sponges or symbiotic microorganisms produce these compounds or if these molecules function in the sponge.

#### (vii) Ctenophora

Ctenophora (comb jellies) is currently considered to be the sister group to all other extant metazoans [129,130]. Comb jellies contain multiple cell types, but lack genes involved in bilaterian mesoderm development [129] as well as many components of canonical stem cell and cancer pathways including the TGF-β, Wnt, hedgehog, fibroblast growth factor and notch pathways, and completely lack the JAK/STAT pathway[129]. Studies of diseases in Ctenophora have reported no tumours [88,131].

### (b) Choanoflagellata

Choanoflagellates are the unicellular organisms most closely related to metazoans [132]. They can form colonies (simple multicellularity). To date, cancer-like phenomena and cheating have not been reported and appear to be understudied in these organisms.

### (c) Fungi: Ascomycota

The sac fungi, Ascomycota, contain over 64 000 species [133] and include complex multicellular forms, such as truffles, morels and cup fungi, as well as simple multicellular and single cell forms, such as powdery mildew, ergot, lichen symbionts, and brewing and baking yeasts. Fruiting bodies in the sac fungi develop from hyphae with single nuclei. In the orange bread mould, *Neurospora crassa*, over 400 genes have been identified that influence the development of the fruiting body. Mutations in several of these cause abnormal vegetative growth [134]. Similarly, at least five different loci [135,136] produce a ‘fluffy’ phenotype of *Aspergillus nidulans*, which is characterized by a rapidly growing mass of undifferentiated hyphae that fail to respond to growth inhibitors and tend to invade and overgrow neighbouring colonies in culture [137]. This fluffy phenotype suggests proliferation inhibition cheating resulting in rapid growth, division of labour cheating in the presence of undifferentiated cells and an invasive phenotype that entails environmental destruction. The presence of an invasive phenotype in Ascomycota means that it should be considered cancerous according to most definitions, though it should be noted that metastasis has not been observed. How the 400 genes involved in the fruiting body interact with environmental factors, such as nutrient deprivation, to influence this phenotype has received extensive study [138]. As in the Basidiomycota, a RAS homologue has been implicated as a key signalling molecule involved in fruiting body formation of *Aspergillus* [139,140] and *Neurospora* [141].

### (d) Fungi: Basidiomycota

**Figure 5.**
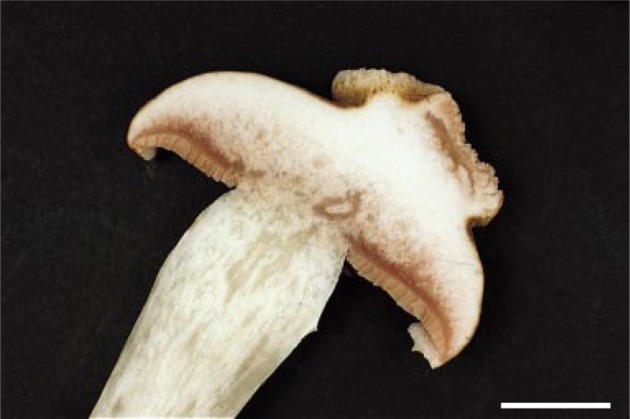
Cancer-like phenomena in basidiomycete fungi. A cross section of the mature fruit body of a commercial mushroom, *Agaricus bisporus.* The abnormal growth demonstrates a cancer-like phenomenon with inappropriate cell differentiation. This is also known as rosecomb disease. Adapted with permission from Umar & van Griensven [149].

This phylum contains over 31 000 species and includes forms with complex multicellular fruiting bodies, such as mushrooms, puffballs and bracket fungi, as well as simple multicellular forms, such as smuts, rusts and *Cryptococcus* yeasts [133]. In contrast to the Ascomycota, fruiting bodies develop from filamentous cells containing two nuclei (dikaryotic hyphae). Mutation screens in several species have identified genes that arrest development prior to fruiting body maturation and spore production [142–144]. In some cases, mutants enable monokaryotic hyphae to proliferate and initiate fruit development [145] or to exhibit one of two cancer-like growth forms. In one type, hyphae form undifferentiated mounds, mats or bulbous forms in *Schizophora commune* [146,147] and in *Coprinus macrorhizus* [148]. Similar growth forms have also been described in cultivated mushroom, *Agaricus bisporus* (figure 5) [149], and associated with genetic alterations including chromosomal modifications [150]. Mound mutants of *S. commune* can be 10 times the size of normal fruiting bodies and can cause neighbouring fruiting bodies to degenerate before overgrowth occurs [147]. The second type of growth form involves inappropriate tissue differentiation, such as gills forming above, instead of below, the fruiting body cap [149]. Both types of abnormal growth interfere with or prevent spore production. Abnormal growth has been hypothesized to arise when cell signals fail to diffuse radially from a central region [149]. Recent studies have demonstrated that fruit formation is influenced by *ras-1* [151,152], a well-known oncogene [153]. Despite this evidence of cancer-like phenomena in Basidiomycota, no cases of cells invading existing tissues have been reported.

The presence of large masses in Basidiomycota indicates proliferation inhibition and/or controlled cell-death cheating, and the lack of differentiation is indicative of division of labour cheating. However, these may fairly be classified as either non-malignant neoplasms or malformations given that invasive cancers have not yet been reported in Basidiomycota.

### (e) Amoebozoa: slime moulds

While most Amoebozoa are unicellular, cellular slime moulds in the genus *Dictyostelium* can be found in both unicellular and multicellular forms and have been extensively studied as a model for the evolution of simple multicellular life cycles and developmental cheating. Specifically, when individual amoebae exhaust their bacterial food supplies, they aggregate to form a slug, the component cells of which contribute to form a stalk (i.e. the soma) or fruiting body (i.e. the germ line). Several elegant studies have shown how chimerism is associated with facultative cheating in the typical organization and differentiation into six cell types [154] occurring during development (reviewed in [155]), whereby amoebae from different clones differentially assort as stalk and spore cells. Interestingly, this case of division of labour cheating seems to be mediated through spatial organization, with cells not producing adhesion factors ending up towards the back of the slug, making them more likely to become part of the fruiting body and differentiate into spores [156], which can be costly for the fitness of the chimeric slug (e.g. [157]). It may be the case that division of labour cheating is mediated through spatial organization of cells more generally, and this spatial organization can be altered by cheating in the production of factors that create and maintain an extracellular environment. This example suggests that cooperation in the creation and maintenance of the extracellular environment is not unique to complex multicellularity. Even social amoeba that are facultatively multicellular create extracellular adhesion factors that lead to a cooperative phenotype and absence of these adhesion factors can provide a fitness benefit for cheaters. The prevalence of these cheating strategies in slime moulds in nature is not known.

Because cellular slime moulds aggregate to form a multicellular chimeric ‘body’ made up of different cell lineages, rather than develop from a single cell, a cheater may not be closely related to other cells, and so might be considered a parasite rather than a neoplasm. Furthermore, cells maintain the ability to leave a particular ‘body’, which potentially provides an alternative mechanism to avoid cheating. Examining cancer-like phenomena in organisms with aggregative multicellularity, such as cellular slime moulds or some bacteria (see below), offers a way to determine which cancer-like phenomena are unique to complex multicellularity.

### (f) Green algae: Embryophyta (plants)

Plants exhibit a variety of cancer-like phenomena, including galls and fasciations. Plant neoplasms can be caused by insects, bacteria, traumatic damage to growing tips, irritation at wound sites or spontaneous mutations (reviewed in 158]). Galls are often induced by bacteria, viruses and insects [159,160], although after initiation altered cells appear to be capable of continuing to proliferate without the continued presence of bacteria [161]. Galls consist of rapidly proliferating and/or disorganized cells, often starting at or near the soil line [159], suggesting proliferation and division of labour cheating. Initially they can appear similar to a callus, but proliferate more quickly [159]. The presence of galls can lead to death and decay of cells on the periphery of the tumour, partially as a result of inadequate resource supply as the gall grows without developing the proper vascular system for delivery of water and nutrients [159], suggesting resource allocation cheating. Galls can either appear as enlarged areas of growth on the plant or can be largely separate, connected by a thin neck of tissue [159].

**Figure 6.**
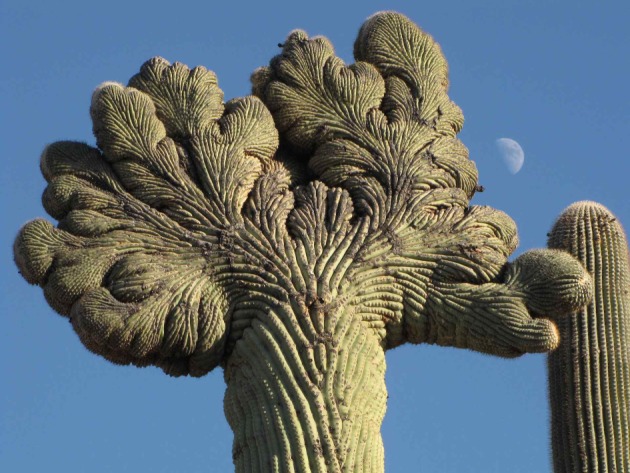
Fasciations in plants. Plants exhibit inappropriate growth patterns known as fasciations thought to be due to somatic mutations in their stem cells. Fasciations in cactus are known as crested cacti and are often botanically desirable (creative commons licence).

Fasciations typically involve the expansion of the main stem axis and can result in a broad and flattened phenotype [162]. Fasciations typically result in uncontrolled and disorganized tissue growth as well as an increase in the amount of tissue [163]. The massive tumour burden suggests proliferation inhibition and/or cell-death cheating and the disorganized nature of that growth probably results from division of labour cheating. They are found in more than 100 plant families [164] and are most common in vascular plants, seed plants and ferns [162]. Fasciation or cristation (cresting) is common among cacti (figure 6), being present in over 50 genera [162,165]. Crest formations have been observed most commonly in older cacti, suggesting that these plant neoplasms could be diseases of ‘old age’ caused by somatic mutations, viruses or trauma [162]. Cacti with crests have become botanically desirable due to the striking formations that can result from fasciation.

Plant tumours can be the result of somatic mutations during development. Mutations are thought to be one cause of cresting in cacti, as many will ‘breed true’ [162]. Other plant cancers are a result of interspecific crosses, resulting in a profusion of tumours on all parts of the plant after the hybrid has reached maturity (as reviewed in [158]). Plant tumours are characterized by both abnormal proliferation and large cell size [166], suggesting that proliferation inhibition and resource allocation cheating may contribute to these growths.

Plant tissue architecture and development differs from animal tissue architecture and development in ways that are likely to be important for cancer suppression. In fact, some have argued that plants are not particularly susceptible to cancer because plant tissue architecture and development keeps cells fixed in place within the cell wall matrix, constraining the capacity of neoplastic plant cells to freely travel through plant tissue in order to invade and metastasize [167]. However, some papers have reported metastasis-like phenomena in plants, with tumour strands emanating from primary plant tumours [166]. Bacteria-free tumours have also been reported at secondary sites without the apparent presence of tumour strands [159]. This suggests that neoplasms in plants may indeed be capable of invasion and establishment of a tumour at a new site (without the presence of bacteria), though there is a need for systematic work in this area.

### (g) Green algae: Chlorophyta

Ulvophyceae, one of the major classes of Chlorophyta, contains species that range from small single-celled organisms to large marine multicellular macroalgae [168], such as sea lettuce. In Chlorophyta, no cancer-like phenomena have been described, though this may be from lack of study. Compounds from the edible sea lettuce (*Ulva fasciata*) have been extracted to test as potential treatment for human cancers due to their antioxidant properties [169].

The Chlorophyceae are another class of Chlorophyta that contains unicellular, colonial and filamentous forms [8]. One of the best known genera is *Volvox*, which forms multicellular colonies made up of many thousands of cells. They are relatively simple in that they possess two primary cell types, large germ cells for reproduction isolated from the environment and small flagellated somatic cells that enable movement of the colony. *Volvox* also exhibit PCD and intracellular communication. Two different types of cancer-like phenomena have been observed in *Volvox*. Somatic regenerator (Reg) mutants begin with both small somatic and large germ cells, but eventually the small somatic cells enlarge and redifferentiate into germ cells. Mutations in genes that repress chloroplast biogenesis are involved, suggesting that the soma/germ line distinction may be maintained through suppressing resource supply and subsequent growth [170]. The other type of cheating occurs in GIs/Reg double mutants in which all cells are initially small, but then redifferentiate into germ cells. These mutants are thought to represent a more ancestral phenotype [170]. Both mutants are consistent with cheating on division of labour or resource allocation.

### (h) Red algae: Rhodophyceae

**Figure 7.**
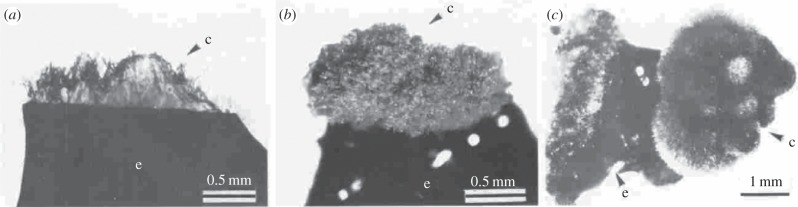
Tumour-like calluses in red algae. Calluses in red algae can take on a variety of morphologies, including disorganized masses, illustrated here in (*a*) *Ptilophora subcostata*, (*b*) *Carpopeltis affinis* and (*c*) *C. prolifera*. Calluses are marked with a ‘c’; the explant (tissue of red algae) is marked with an ‘e’. Adapted with permission from Huang & Fujita [172].

The red algae (which include a variety of seaweeds) were the first eukaryotes to evolve complex multicellularity, around 1.2 billion years ago [36]. They now include 2500–6000 extant species [171]. They are characterized by the accessory photosynthetic pigments phycoerythrin, phycocyanin and allophycocyanin arranged in phycobilisomes, and the absence of flagella and centrioles [171]. Red algae develop cancer-like masses of cells called calluses, though unlike calluses in plants, red algae calluses can retain some structure. Calluses can take on a variety of morphologies, including filamentous, oval and spheroid cell chains, as well as disorganized masses, and can entirely take over the algae in culture (figure 7) [172]. The growth of a mass implies proliferation inhibition and/or controlled cell-death cheating, while the lack of organization within those masses suggests division of labour cheating. Calluses can be caused by abrasions, which were observed in 4% of turbulent-water cultures [173]. Interestingly, normal algae can often be regenerated from a callus. Their formation is facilitated by the inclusion of growth factors that stimulate cell division in culture [174,175]. Tumours can also be generated by exposure to pollutants [176].

Bacteria can also cause galls in red algae, composed of proliferating cells that divide indefinitely [176,177], suggesting proliferation cheating. Like a cancer, these often become ulcerated. They can metastasize in a linear pattern, with hyperplastic tissue between. However, the metastases from these galls also include bacteria and appear not to have evolved independence from the pathogen [178]. This invasive pattern suggests cheating through destruction of the extracellular environment. Localized hypertrophy and hyperplasia can also be caused by a fungus (*Eurychasmidium tumefaciens*) [176]. In addition, there is evidence of gall formation in red algae, in the absence of bacteria and fungus, potentially through viral infection [179].

### (i) Brown Algae: Stramenopiles

**Figure 8.**
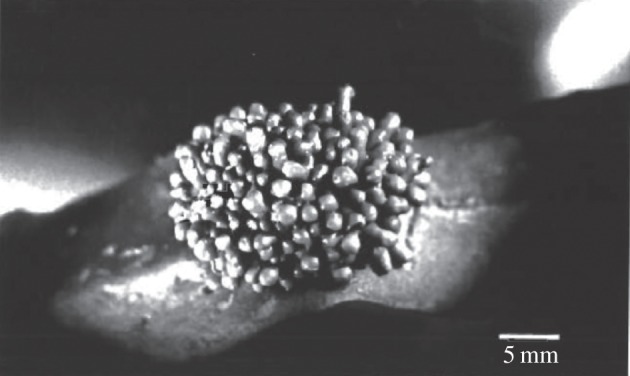
Cancer-like growths in brown algae. Galls or tumour-like growths have been reported in brown algae. Here a frond of *Stephanocystis osmundacea* (formerly known as *Cystoseira osmundacea*) has a multi-pronged gall associated with an *Haloguignardia* fungal infection. Adapted with permission from Apt [181].

Stramenopiles (also called Heterokonts) are in a monophyletic group that includes several lineages with unicellular, colonial, filamentous or complex multicellular forms [8,180]. The most conspicuous multicellular stramenopiles are the marine brown algae (class Phaeophyceae), such as *Sargassum* seaweed and *Laminaria* kelp. Species can vary dramatically in size between microscopic and tens of metres in length with some being commercially important for food and fertilizer. Several species of brown algae have been observed to form galls or tumour-like growths (figure 8) [181], suggesting proliferation and/or cell-death cheating. Calluses of dedifferentiated cells form spontaneously in 2–27% of cultures [182], regardless of media conditions tested [183], indicating division of labour cheating. Actinophryidae may aggregate into a cysteous state, Pelagophyceae can form attached, filamentous, palmelloid and sarcinoid masses, and Phaeothamniophyceae can form filamentous, pseudofilamentous, coccoid or capsoid structures. Whether or not these tumours are caused by somatic mutations is unknown.

### (j) Bacteria

Many bacteria form aggregative multicellular forms (including biofilms, filaments and fruiting bodies), and some bacteria even have post-division cell adhesion (e.g. cyanobacteria and streptomycetes), making them examples of clonal multicellularity. Bacteria that form these multicellular groups exhibit all of the foundations of multicellular cooperation, including controlled cell death, resource transport, division of labour and maintenance of an extracellular environment [184]. They also show proliferation inhibition under stress (including nutrient deprivation and the presence of oxygen free radicals), though it is not clear the extent to which this proliferation inhibition serves the fitness interests of the larger aggregation [185] or simply individual level adaptations to stress. Contact-dependent growth inhibition (a common cancer suppression mechanism in cancer tissues) has been discovered in a variety of bacteria and appears to be involved in biofilm formation and other multicellular cooperative phenomena [186], though again it is unclear whether this has been selected as an individually or collectively beneficial phenotype.

Several bacterial species that form aggregations have been extensively studied as models of cellular cooperation and cheating (but see e.g. [187,188]). Some of these, such as the production of siderophores, iron-chelating molecules produced by certain bacterial species to sequester otherwise unavailable iron, are forms of collective resource acquisition. Others, such as the creation and maintenance of the extracellular environment in the form of biofilms, serve the dual function of resource access and defence, because it allows colonization of air-liquid interfaces and provides protective structures against predation and toxic molecules. Cells may emerge from within biofilms, or migrate from the outside into these aggregations, and may either contribute to the quality of the extracellular environment (i.e. ‘cooperate’) or ‘cheat’, whereby the benefits of other cooperators are accrued at a reduced personal cost associated with not contributing to the public good. For example, motile bacilli can penetrate and kill heterologous biofilms [189]. Similarly, studies on the rhizosphere bacterium *Pseudomonas fluorescens* SBW25 have shown that insufficient regulation of cheating strategies may result in lowered group fitness (e.g. [190,191]). Overgrowth in bacterial biofilms and cheaters in siderophore producing populations are not known to exhibit regulatory cheater control. Without such control, faster growing lineages can over-exploit resources, resulting in a population crash. Regulatory control resulting in population persistence could theoretically emerge if there is a trade-off between growth rate and yield. Evidence for proliferation control in bacteria was found in experiments on *Salmonella enterica* (serovar Typhimurium) [192]. Finally, similar to *Dictyostelium discoideum* slime moulds, the bacterium *Myxococcus xanthus* has been shown to exhibit division of labour cheating, with colonies typically formed by an aggregation of different genotypes [193,194]. Together these results suggest that organisms that exhibit aggregative multicellularity exhibit both cooperation and cheating in several of the foundations of multicellularity. However, to our knowledge there have been no studies of cancer-like phenomena in clonally multicellular bacteria such as cyanobacteria.

## 4. Discussion

### (a) Generalizing cancer across life

Cancer has been recognized and defined anthropocentrically by how it appears in humans (and to a lesser extent, laboratory mice). In order to recognize and understand cancer across multicellular life forms, we described five aspects of cooperation that are necessary to maintain a multicellular body: (i) proliferation inhibition, (ii) controlled cell death, (iii) division of labour, (iv) resource allocation and (v) extracellular environment maintenance, which we term the five foundations of multicellularity. Cancer and cancer-like phenomena in any multicellular organism can then be seen as cheating on one or more of these features of cooperation.

Given our definitions, animals appear to be more susceptible to cancer than the other branches of multicellularity, although we cannot completely eliminate the possibility that this conclusion is influenced by sampling bias as there have been vastly more studies of cancer in animals than in other organisms. Moreover, that cancer is observed throughout the Eumetazoa, does not mean that it is common in any particular species. Most observations of zoo animal species indicate cancer incidences of less than 5% [52]. Nevertheless, cancer susceptibility in animals can be related to diverse aspects of cooperation. Animals require greater division of labour, represented by the greater number of cell types in animals compared to plants, fungi and algae [37]. Higher metabolic rates in animals relative to plants, fungi and algae provide the resources required for cooperative distribution systems and perhaps also facilitated the evolution of more cell types [37]. Higher metabolic rates might, therefore, leave animals more susceptible to resource allocation and division of labour cheating as well as increase the cancer risk directly [11]. In addition, cancer susceptibility in animals may be due to a larger number of proliferative cells in some epithelial and immune tissues in adult animals, relative to other forms of multicellularity. Proliferating cells are susceptible to accumulating somatic mutations leading to the cellular evolution that drives carcinogenesis. Animals also have circulatory systems that transport cells, as well as resources, which probably make them more susceptible to metastasis than organisms that only transport resources. Cell migration and metastasis are also likely to be more difficult to evolve in organisms with cell walls [195]. Aside from a few rare observations in plants [159,196], metastasis appears to be restricted to animals.

Contrary to conventional wisdom, both cnidarians (coral) and plants exhibit cancer-like phenomena. The observation of cancers in cnidarians, which are a sister group to Bilateria, suggests that some of the cancer susceptibility of bilaterian animals may have origins in genetic and physiological changes that occurred in their common ancestor. Cnidarians have been considered the most basal lineage to evolve gap junctions [7], which may lead to cancer susceptibility. We further speculate that plants may be particularly susceptible to cancer-like growths because of their large size, longevity (compared to sister groups), and peripheral stem cell distribution in which somatic mutations can accumulate owing to the large number of cell divisions. Cancer-like phenomena appear to be less lethal in plants than in animals, and it may even be the case that some genetic instability enables morphological variants (but also could lead to susceptibility to cancer-like growths), which might provide survival advantages to plants in stressful environments.

Our survey of cancer-like phenomena across the tree of life (figure 2) reveals that cheating on some forms of cooperation is more common than for others. For example, cheating of proliferation inhibition and/or regulated cell death was apparent in all branches of multicellularity exhibiting cancer or cancer-like phenomena, though it was often difficult to distinguish which form of cheating led to a tumour mass. Division of labour cheating, observed as undifferentiated or disorganized cell masses (including ‘lesions'), was also present in all forms of multicellularity, suggesting that it may be an important and perhaps underappreciated form of cheating in multicellularity. Cheating in resource allocation and maintaining the extracellular environment have only been documented in animals and plants so far, suggesting that increased complexity may come at the price of further susceptibility to resource allocation and environmental maintenance cheating in multicellular bodies. The apparent absence of resource and environment cheating in fungi, as well as red and brown algae, may be due to their absence, but may also be due to insufficient study of those organisms and the relative difficulty of assessing those forms of cheating.

Given these observations we suggest that it may be useful to distinguish between two categories of cheating that occur in multicellular organisms and lead to cancer-like phenomena: demographic (including proliferation and cell-death cheating) and economic (including resource, labour and environment cheating). We found that both types of cheating occurred in each report of cancer or cancer-like phenomena in our review of the literature. Cheating of both types may be necessary for cancer/cancer-like phenomena. Future work should further identify and distinguish the necessary and sufficient forms of somatic cheating that lead to cancer and cancer-like phenomena.

Cancer phenotypes associated with demographic cheating have long been considered central to carcinogenesis [9,10]. Economic cheating, on the other hand, has been less well studied. There are likely to be many unrealized opportunities for applying the vast literature on cooperation theory and social evolution to economic cheating within neoplasms. For example, phenomena such as public and private good production [197], positive assortment [198], policing and punishment [199] are likely to play important roles in economic cooperation in multicellular bodies just as they do in other complex social systems.

Demographic and economic cheating differ with regard to clinical presentation and the evolutionary/ecological dynamics underlying them. Demographic cheating presents clinically as a tumour mass, since proliferation and a lack of cell death both lead to an increase in the number of cells. Economic cheating has more diverse clinical manifestations, including a lack of differentiation (labour cheating), invasion of blood vessels (resource cheating) and necrosis (environment cheating). Another important difference between the two types of cheating is that demographic cheating is most relevant for the evolutionary dynamics of the tumour (since proliferation and apoptosis affect the population composition) while economic cheating leads to changes in the ecological dynamics through effects on the tumour environment. Economic cheating without demographic cheating might manifest in abnormal cell differentiation, resource monopolization (including large cell size) and degraded environmental conditions in the tissue but no tumour. Demographic cheating without economic cheating could lead, however, to other pathological conditions such as developmental defects or metabolic disorders. Further investigation into the role of demographic cheating in other aspects of health and disease (e.g. metabolic disorders) may be a productive direction for future research.

### (b) Cancer suppression is required for complex multicellularity

Cancer suppression includes both the enhancement of cooperation and the suppression of cheating. From this perspective, complex multicellularity represents a highly sophisticated cheater detection and suppression system. Organisms have evolved a variety of mechanisms to suppress somatic cheating. Some forms of cancer suppression are cell autonomous, such as an apoptotic response to DNA damage or inappropriate proliferation. Others are structured by the tissue architecture, such as sequestration of rare stem cells [200], or systemic, such as immune surveillance. Some of these cancer suppression mechanisms are shared because they evolved in a common ancestor. Others seem likely to have evolved separately because they occur in disparate lineages. Thus, novel suppression mechanisms may be found through investigation of organisms that have reduced cancer incidence. For example, the absence of cancer-like phenomena in hemichordates, placozoans, sponges, ctenophores and chlorophytes (Ulvophyceae), suggests that important research remains to be done to document and explore the extent of their resistance to cancer. A simple and feasible approach to this could include studies of carcinogen or radiation exposure to organisms that can be maintained in the laboratory.

To develop and survive, large and complex organisms require continual cooperation among cells, tissues and organ systems while suppressing cheating. One partial solution to the problems that arise in scaling-up multicellularity may be clonal multicellularity, i.e. developing from a single cell zygote. This reduces conflict and enhances the likelihood of the evolution of cooperation, which makes large-scale and complex multicellularity more viable. However, aggregative multicellularity, such as bacterial biofilms, can also exhibit large-scale cooperation and surprisingly complex organization [201,202], suggesting that clonality is not strictly necessary for cooperation and complexity in multicellular groups of cells.

Another potential solution to the problem of suppressing cheating as multicellularity scales up to large body sizes may be the organization of some tissues into proliferative units (e.g. intestinal crypts), with a small number of stem cells and their differentiated progeny [203]. Many epithelial tissues in vertebrates are organized into these proliferative units, which have been proposed to exert some within-unit control over proliferation [204,205]. Subdivision of the population of somatic cells in this way may enable regulation of cooperation both within and between these units, breaking down a potentially unmanageable cheater suppression problem in a large multicellular body into more manageable and modular sub-problems. If mutant stem cells have difficulty expanding beyond the proliferative unit owing to either suppression within the unit or cheater suppression systems operating between units, this tissue subdivision could function as a powerful cancer suppression mechanism [200]. Another potential benefit of enforcing multicellular cooperation at the level of proliferative units may be slowing the speed of evolution and therefore cancer progression. If proliferative units (rather than cells) act as the unit of selection within the body, the effective population size (and speed of somatic evolution) would be reduced by several orders of magnitude, and the generation time would lengthen to the generation time of the proliferative units. Proliferative units may therefore form an intermediate and little studied level of selection between the cell and organism level. They also exhibit many of the foundations of multicellular cooperation and individuality: they exhibit proliferation inhibition, extracellular environment maintenance and can reproduce as a unit through the process of crypt fission [206]. Together, these characteristics of proliferative units suggest that the literature on levels of selection (e.g. [207,208]) may be relevant to proliferative units within somatic tissues. Future work to characterize the dynamics within and between proliferative units should help elucidate the levels at which selection is taking place and the role proliferative units may play in facilitating the evolution of large tissues and body size.

Studying organisms that get dramatically less cancer than expected may provide important insights into cancer suppression as well. Discovery of nature's cancer suppression mechanisms may suggest methods for better cancer prevention in humans. Elephants have approximately 100× more cells than humans, and whales have approximately 1000× more cells (with similar lifespans to humans), but do not get proportionally more cancer (Peto's paradox) [11]. The focus of work on Peto's paradox has been on the number of cell divisions and opportunities to accumulate mutations. However, large organisms also require better resource transportation and allocation as well as better maintenance of the extracellular environment than smaller organisms. Across multicellular life, larger organisms have more cell types and thus more division of labour than smaller organisms [37]. Whether this is true across mammals is not known [209]. Division of labour, resource transport and environment maintenance might enable more effective large-scale multicellularity but leave an organism vulnerable to cheater cells (i.e. cancer). Thus, large organisms are likely to require greater cheater detection and suppression than small organisms, across all the foundations of multicellularity, in order to generate and maintain functioning multicellular bodies. One common hypothesis to resolve Peto's paradox is that large organisms have evolved more checks on carcinogenesis (cheater suppression mechanisms) than smaller, short-lived organisms. Elephants appear to have evolved many copies of the important tumour suppressor gene TP53 [11] and may employ additional, and as yet unknown, methods to prevent cancer. The cancer suppression mechanisms in whales are currently unknown.

Finally, several lines of evidence indicate that the apparent absence of cancer in both the naked mole rat and blind mole rat is related to enhanced multicellular cooperation and suppression of cheating. First, exceptionally sensitive contact inhibition capacities leads to greater proliferation inhibition in the naked mole rat [210] and secretion of interferon-β1 induces necrosis of neighbouring cells in the blind mole rat [55]. Second, possession of a version of hyaluronan (a component of the extracellular matrix) that appears to confer high levels of cancer suppression in the naked mole rat [211] and an alternatively spliced form of heparanase that represses heparan sulfate degradation at the cell surface and in the extracellular matrix in the blind mole rat [212] suggest that these animals have adaptations for protecting the extracellular environment. Finally, due to their hypoxic environment, naked mole rats exhibit low metabolic rates with unusual capacities for metabolic regulation [213], which may be an indication of cooperative resource allocation among cells. Thus, three of the five foundations of multicellular cooperation (proliferation inhibition, maintenance of the extracellular environment and resource allocation) may be enhanced in naked and blind mole rats. In addition to their hypoxic environments, naked mole rats and blind mole rats have adapted to relatively low levels of predation and, in the case of the naked mole rat queen, fertility that does not decrease with age [214]. These selective pressures likely select for longer lifespans and better somatic maintenance.

Future research should focus on the kinds of information and mechanisms that could be used by a multicellular body to detect and eliminate somatic cheaters, whether it is the composition of the stroma, the shape of cheater cells or proteins expressed by them, or some other form of aberrant signals generated by the neoplastic cells. This approach should lead to a series of experimentally testable hypotheses identifying the mechanisms of cancer suppression in organisms, which may be enhanced and used for cancer prevention in humans. Cancer is not just a matter of cells that are inherently cheaters, but also a matter of being in an environment that has poor cheater detection and suppression. Work on the cancer microenvironment is therefore also relevant and important to understanding the ways in which somatic cheating is suppressed.

### (c) Cheating and cooperation within neoplasms

#### (i) Cheating may enhance selection for invasion and metastasis

A tumour which is not yet invasive or metastatic can often be surgically removed and the patient can be cured. Understanding the causes of invasion and metastasis is therefore a central problem for cancer treatment and prevention. Cell motility is one important aspect of cell phenotype that enables invasion and metastasis. Models have shown that cheating in resource allocation within neoplasms can lead to selection for cell motility [215]. Cheating in the other foundations of multicellularity may result in dispersal evolution as well, as high density (resulting from proliferation or cell-death cheating) and poor conditions (resulting from resource, labour or environment cheating) generally lead to selection for the ability to leave degraded environments. In other words, the presence of somatic cheating may alter the environment and selection pressures on cells so as to favour motile phenotypes. These motile cells might then be under further selection for cheating since mobility can in some circumstances favour cheating [216]. Cancer progression might therefore be partially a result of a positive feedback where cheating generates selection pressures for dispersal and the resulting motile cells are under even greater selection for cheating within the host.

#### (ii) Cooperation within neoplasms

The focus of this review has been on cancer as cheating in the foundations of multicellularity, but another important potential application of this framework is cooperation within neoplasms [217–219]. Recent work suggests that cooperation among cancer cells occurs [218,219] and that metastases are often the result of aggregations of cells rather than single cells [220]. In this section we briefly explore the possibility that neoplasms recapitulate some of the foundations of multicellular cooperation, benefiting the colony of cancer cells rather than the host. Multilevel selection can lead to selection for cooperation when a population is made up of distinct and genetically diverse groups [221,222]. In advanced disease, metastases can form a metapopulation with genetically diverse colonies of cells [223], leading to the kinds of population structure that could favour cooperation within the cell colonies [224].

Many of the phenotypes observed in advanced cancer suggest that the foundations of multicellular cooperation may re-emerge in later stages of progression. For example, the phenomenon of ‘tumour dormancy,’ where micrometastases appear to exhibit no growth for extended periods of time [225] may be the result of proliferation inhibition or the regulation of cell death within those micrometastases which could indicate a recapitulation of these demographic forms of multicellular cooperation within the tumour. During progression, tumours evolve the capacity to effectively create a vascular network to supply the tumour with oxygen and nutrients, using the same angiogenic signals that the multicellular body uses to create the vascular systems [226]. This promotes the supply and distribution of resources as well as the removal of wastes [226], suggesting that vascular signalling might represent some form of resource allocation cooperation and perhaps also environment maintenance cooperation within tumours. With regard to the final foundation of multicellular cooperation: models have shown that reproductive division of labour can evolve within neoplasms with certain cells acting as a kind of ‘germ-line’ of the tumour and perhaps corresponding to what cancer biologists have observed as ‘cancer stem cells' that are capable of recreating a tumour, and other cells acting as a ‘somatic line’ by limiting their own proliferation and enhancing the fitness of the ‘stem-like’ cells [227]. Together these findings suggest the possibility that cancer progression can be characterized by the evolution of new ‘protomulticellular’ entities inside the host that recapitulate the foundations of multicellular cooperation.

### (d) Relevance for comparative oncology, cancer prevention and management

**Figure 9.**
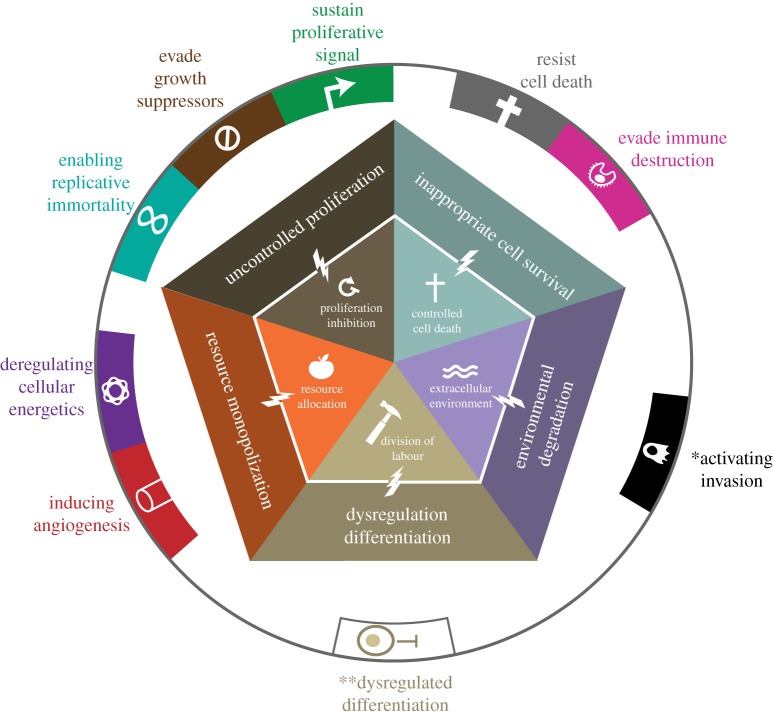
Foundations of multicellularity and cancer hallmarks. The hallmarks of cancer correspond closely to cheating in the foundations of multicellular cooperation. However, there are no currently recognized hallmarks that correspond to the breakdown of the division of labour in a multicellular body, suggesting that dysregulation of differentiation (**) may be a missing hallmark. Also, the hallmark of invasion/metastasis maps incompletely onto this framework (*). Invasion is partly a result of degradation of the extracellular environment, but metastasis is a more complex process that may require cheating in many of the foundations of multicellularity.

The current paradigm for understanding the characteristics of cancer is the ‘hallmarks of cancer’ approach, which was developed from observations of common phenotypes across human cancers [9,10]. Each of the hallmarks of cancer corresponds to cheating in the foundations of multicellularity, but not every feature of multicellularity relates to a cancer hallmark (figure 9). Cheating in division of labour is not represented in the hallmarks. This type of cheating could manifest clinically as a lack of cell differentiation or inappropriate cell differentiation. Cheating in division of labour appeared in all cancer and cancer-like phenomena in our survey of cancer across the tree of life, indicating that dysregulated differentiation may be central to cancer across life. Disruption of differentiation is also ubiquitous across human cancers [39]. It is the basis of much of the pathological grading of cancers, and there is theoretical reason to believe it is a central and early event in carcinogenesis [228,229]. Though the importance of the disruption of differentiation is widely recognized in cancer biology, it has been subsumed within the insensitivity to growth signals hallmark [9]. Differentiation, often called ‘terminal differentiation,’ involves exiting the cell cycle for most cell types. However, exceptions, such as beta cells in the pancreas with the capacity to self-renew [230] and hepatocytes and cholangiocytes, the two epithelial cell types of the liver that can function as facultative stem cells for each other [231], show that differentiation and growth suppression are formally separate phenotypes. Our analysis of the foundations of multicellularity suggests that division of labour should be treated separately from proliferation inhibition. Together these observations suggest that dysregulated differentiation resulting from division of labour cheating may be a missing hallmark of cancer.

One other exception to the alignment of the foundations of multicellularity and the cancer hallmarks is metastasis. In the hallmarks framework, invasion and metastasis are grouped together as a single hallmark. However, only invasion aligns with the foundations of multicellularity framework: invasion is a consequence of degradation of the extracellular environment, while metastasis requires many additional cell-level capacities to effectively reach and colonize new environments. Metastasis might be facilitated by cheating many of the foundations of multicellularity: proliferation and cell-death cheating fuelling population growth in new metastases, division of labour cheating facilitating dedifferentiation, resource cheating feeding the metastasis at the expense of the host, and environment degradation enabling invasion into new tissues and organs. As discussed earlier in this paper, invasion and metastasis might both be a result of the effects of cheating on the degradation of the environment and subsequent selection for dispersal from that environment. Finally, metastasis might also be characterized by the rebuilding of some of the foundations of multicellular cooperation within the neoplasm in ways that could facilitate growth in new tissues and the release of viable propagules (micrometastases) [224].

We propose that the foundations of multicellularity provide a framework for analysing the state and progression of a neoplasm that can be generalized across all forms of multicellular life. We further suggest that cancer management might benefit from classifying the forms of cheating present and targeting those forms of cheating. One consequence of this perspective is that mechanisms that underlie each of the forms of cheating in the foundations of multicellularity could be related to clinically measurable features of neoplasms. For example, resource allocation cheating and maintenance of the shared environment cheating can be generated by the mechanism of switching to a glycolytic metabolism (the Warburg effect), because glycolytic metabolism dramatically increases glucose uptake and its by-products degrade the local environment. Glucose uptake can be measured non-invasively through positron emission tomography (PET) scan technology.

Other forms of cheating are achieved through altering signal production or reception. For example, proliferation inhibition cheating can be caused by inappropriate generation of growth factors or by suppression of anti-growth receptors. Production of survival factors, the epithelial-to-mesenchymal transition, and evasion of the immune system (by a variety of alterations) are all common mechanisms of cheating observed in neoplasms. Mechanisms for cheating in these and other foundations of multicellularity can generally be assayed using standard techniques (table 1).

**Table 1.** Summary of types of cheating on each multicellularity foundation with clinical phenotype and measurable features.

	multicellularity foundation	type of cheating	clinical presentation	assays for cheating
demographic cooperation	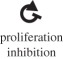	inappropriate proliferation	tumour mass	proliferation assays (e.g. Ki67 [232], PCNA [233], BrdU [233] or EdU [234] incorporation) mitotic index (DAPI [235], H&E [236], etc.) cell-cycle assays (cyclin D1 [237], etc.) competition assays in cell culture [238] assays that preserve tissue architecture (e.g. IHC [239] or mitotic figures in fixed tissues [240]) can reveal proliferation in inappropriate locations
		insensitivity to anti-growth signals	tumour mass	anti-growth receptor assays (e.g. by IHC) anti-growth signal cascade assays [241] expression assays for growth regulatory genes (e.g. RNA-Seq [242])
		disruption of senescence	tumour mass	senescence assays (e.g. beta-gal [243], telomerase [244])
	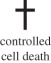	suppression of PCD	tumour mass	PCD assays (e.g. Caspase 3, 6 or 7, Annexin V, TUNEL, Propidium iodide, cytochrome *c*) [245]
economic cooperation	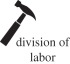	loss of differentiation	undifferentiated cells	tissue architecture (H&E) tissue-specific differentiation markers
		loss of cell function	atrophy, metaplasia	cell-type-specific functional assays expression assays of specialized cell function genes (RNA-Seq)
	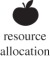	increased resource accumulation	large cells	cell size (H&E, image/flow cytometry [246,247])
		unrestrained metabolism	increased mitochondria	measure the number of mitochondria (e.g. TMRE or TMRM [248]) rate of ATP production [249]
			increased glucose use	glucose uptake (PET scan [250] or stains for glucose transporters) increased expression of metabolic genes (e.g. RNA-Seq)
		inappropriate angiogenesis	neoangiogenesis, abnormally dense microvessels	angiogenesis (stains for endothelial cells and angiogenic factors [251])
	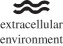	destruction of tissue architecture	loss of basal membrane	basal membrane degradation (H&E, matrixmetalloprotease [252] stains)
		insufficient supporting infrastructure	necrosis	H&E, hypoxia assays (e.g. EF5 [253])
		accumulation of waste products	accumulation of lactic acid	pH monitoring (e.g. ratio imaging microscopy [253])
			accumulation of cell debris	macrophage engulfment of apoptotic cells (e.g. by pHrodo succinimidyl ester [254])
		failure to produce a common good	lack of a common good	cellular production of common good

Basing the assessment of neoplasms on the foundations of multicellularity has the advantage of generalizability across organisms, facilitating comparative oncology. This suggests future studies to test if biomarkers, based on measurement of the different forms of cheating, provide clinical (including veterinary) utility for risk stratification and clinical management of neoplasms.

Understanding neoplastic progression as the progressive evolution of cheating on each of the foundations of multicellularity suggests a research programme in cancer prevention based on the detection (table 1) and suppression of cheaters in these forms of cooperation. This might be achieved through exogenous interventions, or through bolstering the body's endogenous mechanisms for cheater suppression. Furthermore, elucidation of cheater suppression mechanisms in non-human species may lead to novel methods for cancer prevention in humans.

## Endnote

1 Knoll defines complex multicellularity as involving differentiation into more than just two cells types (reproductive and somatic), intercellular communication, often programmed cell death, three-dimensional organization with some cells not in contact with the environment, and resource transport systems to deliver nutrients and remove waste when diffusion is no longer adequate to service all the cells of the body [7].
